# Optimized mtDNA Control Region Primer Extension Capture Analysis for Forensically Relevant Samples and Highly Compromised mtDNA of Different Age and Origin

**DOI:** 10.3390/genes8100237

**Published:** 2017-09-21

**Authors:** Mayra Eduardoff, Catarina Xavier, Christina Strobl, Andrea Casas-Vargas, Walther Parson

**Affiliations:** 1 Institute of Legal Medicine, Medical University of Innsbruck, 6020 Innsbruck, Austria; Catarina.Gomes@i-med.ac.at (C.X.); christina.strobl@i-med.ac.at (C.S.); 2 Grupo de Genética de Poblaciones e Identificación, Instituto de Genética, Universidad Nacional de Colombia, Bogotá, Colombia; lacasasv@unal.edu.co; 3 Forensic Science Program, The Pennsylvania State University, University Park, PA 16802, USA

**Keywords:** primer extension capture, mitochondrial DNA, Massively Parallel Sequencing, forensic science

## Abstract

The analysis of mitochondrial DNA (mtDNA) has proven useful in forensic genetics and ancient DNA (aDNA) studies, where specimens are often highly compromised and DNA quality and quantity are low. In forensic genetics, the mtDNA control region (CR) is commonly sequenced using established Sanger-type Sequencing (STS) protocols involving fragment sizes down to approximately 150 base pairs (bp). Recent developments include Massively Parallel Sequencing (MPS) of (multiplex) PCR-generated libraries using the same amplicon sizes. Molecular genetic studies on archaeological remains that harbor more degraded aDNA have pioneered alternative approaches to target mtDNA, such as capture hybridization and primer extension capture (PEC) methods followed by MPS. These assays target smaller mtDNA fragment sizes (down to 50 bp or less), and have proven to be substantially more successful in obtaining useful mtDNA sequences from these samples compared to electrophoretic methods. Here, we present the modification and optimization of a PEC method, earlier developed for sequencing the Neanderthal mitochondrial genome, with forensic applications in mind. Our approach was designed for a more sensitive enrichment of the mtDNA CR in a single tube assay and short laboratory turnaround times, thus complying with forensic practices. We characterized the method using sheared, high quantity mtDNA (six samples), and tested challenging forensic samples (*n* = 2) as well as compromised solid tissue samples (*n* = 15) up to 8 kyrs of age. The PEC MPS method produced reliable and plausible mtDNA haplotypes that were useful in the forensic context. It yielded plausible data in samples that did not provide results with STS and other MPS techniques. We addressed the issue of contamination by including four generations of negative controls, and discuss the results in the forensic context. We finally offer perspectives for future research to enable the validation and accreditation of the PEC MPS method for final implementation in forensic genetic laboratories.

## 1. Introduction

Mitochondrial (mt)DNA is present in a higher copy number in the cell than nuclear DNA, which is why its analysis can be useful in forensic cases, where the evidentiary material does not contain enough nuclear DNA for conventional autosomal Short Tandem Repeat (STR) typing [1]. mtDNA haplotypes are less informative than combined autosomal genotypes, as they are shared by maternally related individuals and do not undergo recombination. Its meaningful application in the forensic context is therefore restricted to cases that involve (a reduced number of) maternally unrelated individuals. It represents a powerful tool in disaster victim identification [2] and in human identification cases [3,4], particularly when evidentiary and reference samples are separated by multiple generations. Typically, hair (shaft) and solid tissue samples are analyzed with mtDNA and usually associated with high success rates (e.g., [5]). Early protocols targeted the two hypervariable segments of the mtDNA control region (CR), Hyper Variable Segments HVS-I and HVS-II [6], which contain the majority of fast-evolving sites in the mtDNA genome (mitogenome). However, the separate amplification and sequencing of these segments turned out to be prone to sample mix-up (aka artificial recombination) when performed manually [7], which is why the amplification of the entire CR in one fragment [8] and sequencing of the amplicon with internal primers is preferred [7,9]. For degraded mtDNA, smaller amplicon strategies were developed to target ‘medium-sized’ fragments (approximately 300 bp [10]) and ‘mini-sized’ fragments (approximately 150 bp [11,12]) of the mtDNA CR in a PCR multiplex format. These approaches succeeded in avoiding artificial recombination by overlapping amplicons and redundant sequencing coverage, and are considered state of the art. With the emergence of Massively Parallel Sequencing (MPS) technologies, the concept of small overlapping amplicons was meanwhile extended to the analysis of the entire mitogenome and brought useful mitogenomes in the same forensic samples that gave only CR sequences with established Sanger-type Sequencing (STS) and Capillary Electrophoresis (CE)methodology [13,14].

When evidentiary samples are exposed to extreme conditions, such as excessive heat, advanced decomposition, or when they are submerged in water for a longer time period, the mtDNA usually degrades to an extent such that mini-amplicons cannot be generated from the extracted mtDNA. Further reducing the amplicon size might not be an effective solution to this problem, as the region of interest becomes shorter and the primer design more problematic, particularly for highly variable mtDNA regions. For most forensic applications, the mini-amplicon strategy seems to represent the lower limit of PCR-based sequencing approaches and samples that do not provide meaningful results were usually not further investigated.

The fields of archeology and paleontology have pioneered the development of molecular genetic methods that cope with the nature of ancient (a)DNA that is typically present in minute amounts, highly degraded, and chemically modified by environmental conditions [15]. Due to its high copy number, mtDNA was the first genetic marker to be analyzed in aDNA. The rise of MPS-based assays significantly increased the success of obtaining useful genetic data from ancient specimens [16,17], especially by using DNA hybridization enrichment and single-step PCR primer extension capture (PEC) [18]. Probe hybridization assays use 70–500 base pair (bp)-long biotinylated DNA or RNA probes hybridizing to regions in the mitogenome, and are either generated from PCR products [19,20,21,22] or designed and made commercially available (e.g., Mybaits (Microarray, Ann Arbor, MI, USA) [23] or Agilent’s SureSelect (Agilent Technologies, Santa Clara, CA, USA) [24]). However, the protocols usually require high library DNA input (more than 100 ng), and the hybridization steps can take up to 72 h. Single Step Extension Capture was developed by Briggs et al. in 2009 [25] for the sequence analysis of Neanderthal mitogenomes. The protocol uses lower DNA library input (50 ng) and shorter turn-around times, and was adapted in other studies [20,26].

With this study, we offer an adapted PEC protocol having forensic requirements in mind, i.e., targeting the mtDNA CR only (most forensic laboratories worldwide are not (yet) performing coding region (codR) analysis due to legal restrictions). We further optimized the method to provide a single tube assay that requires less DNA extract and only short sequencing and analysis times. The performance of the optimized PEC method is demonstrated by application to forensic and ancient solid tissue samples that provided no or only weak mtDNA data with conventional STS or primer-based MPS methods.

## 2. Materials and Methods

### 2.1. Samples and DNA Extraction and Quantification

In this study, two modern forensic samples (5 and 3 cm hair shafts) and 15 ancient solid tissue samples (six teeth and ten bone samples) were investigated using the PEC method (Table 1). DNA from the hair samples was extracted using the EZ-1 advanced instrument and the QIAamp DNA Investigator kit (Qiagen, Hilden, Germany) following the manufacturer’s recommendations. The solid tissue samples originated from two excavation sites in Volders, Austria [27] and Sogamoso, Colombia, and from three different age categories of approx. 1, 2, and 8 kyrs, respectively. DNA from the solid tissue samples was extracted using the Phenol Chloroform and Spin Filter methods described in [27]. DNA for the positive control and quantification standards was extracted from human blood using the QIAamp DNA Blood Maxi Kit (Qiagen). Extraction blanks and negative controls (no template control (NTC-PEC), no primer control (NPC), enrichment control (ERC), and library preparation blank (NTC-LP)) were included in each batch (details see below). mtDNA copy number was determined via qPCR using a 143 bp target TaqMan assay according to ([28], updated in [29]).

### 2.2. Control Sample Types and Preparation

The positive control samples were prepared in different laboratory facilities and independently from the forensic and ancient solid tissue samples. They were prepared earlier in batches and stored in aliquots at −20 °C. The DNA for the positive controls was sheared for 40 min using the Ion Shear Kit (Thermo Fisher Scientific (TFS) Waltham, MA, USA) to produce degraded DNA. An enrichment control was prepared by amplifying a 405 bp region of the mitogenome of the positive control using two primers at positions 8251 (forward, 5′-GCCCGTATTTACCCTATAGCAC-3′) and 8656 (reverse, 5′-CTCATCAACAACCGACTAATCA-3′). The final products were then quantified using the 143 bp TaqMan assay and diluted to 10,000,000 copies/µL, which corresponds to the Agilent HS DNA chip’s (Agilent Technologies, Santa Clara, CA, USA) detection limit of 5 pg (9,358,197.36 molecules of amplification product). To simulate background DNA, 1 µg *Escherichia coli* DNA (D2001-5MG strain B Type VIII; Sigma Aldrich, St. Louis, MO, USA) was sheared for 30 min using the Ion Shear Kit (TFS). Finally, a total of 15–20 ng of this sheared *E. coli* DNA was mixed with 1 µL of diluted PCR product for enrichment control. After PEC, 1 µL of this enrichment control showed a clear peak at 400 bp on the Agilent HS DNA chip (Agilent Technologies). Library preparation for all controls was performed using the IonXpress Fragment Library Kit (TFS) according to the manufacturer’s protocol. Furthermore, in order to monitor potential human DNA contamination during library preparation, single source *E. coli* DNA was used as a library preparation control. Separate amplification controls of 1 µL sheared positive control DNA were used after each PEC round.

### 2.3. Library Preparation

Library preparation was performed using the IonXpress Fragment Library Kit (TFS) according to the manufacturer’s protocol. DNA extracted from forensic and solid tissue was not subjected to shearing first, but library preparation was done directly from the extract in the same way as described above. After library preparation, all samples were amplified for 10 cycles using polymerase and primer mixes from the Library Preparation Kit according to the manufacturer’s protocol (TFS, details see Table 1). Libraries for analyses with the commercially developed mitotiling (MT) assay were prepared using the Ampliseq technology according to the manufacturer’s protocol (TFS).

### 2.4. PEC Primer Design

A total of 14 primers were carefully designed using Primer3 [30,31] to span the mtDNA CR. Special care was taken to choose primers of similar length, melting temperature, and GC content (Table S1). An additional primer was included that annealed to the region targeted by the previously described 143 bp TaqMan quantitation assay (position 8342 [28]) for initial enrichment quantification purposes and enrichment control. Biotinylated primers were produced by Microsynth (Microsynth AG, Balgach, Switzerland).

### 2.5. Primer Extension Capture Workflow and Reaction Details

This paragraph describes the details and modifications of the PEC workflow originally developed by Briggs et al. [25]. All samples, including positive control (PC), no template control (NTC-PEC), no primer control (NPC), enrichment control (ERC), and library preparation blank (NTC-LP) were treated in the following way: for PEC, 20–25 µL of DNA extract were mixed with 5 µL of 10× Amplitaq Gold Buffer, 5 µL of MgCl_2_ (25 mM), 2 µL BSA (2.5 mg/mL), 1 µL dNTPs (10 mM), 5 µL of PEC Primer Mix (1 µM), and 1.2 µL of Amplitaq Gold DNA Polymerase (5 U/µL, TFS) and topped with bidistilled water to final 50 µL reaction volumes. An initial denaturation step at 95 °C for 12 min was followed by a primer annealing step at 57 °C for 1 min and an extension at 72 °C for 3 min. The reaction was stopped by adding 10 µL of 0.5 M EDTA, mixing, and putting the sample tube on ice. For bead capture of biotinylated primer-DNA-string duplexes, 30 µL of Dynabeads MyOne Streptavidin C1 (TFS) per sample were washed three times with 2× Binding & Wash (BW) buffer according to the manufacturer’s protocol and re-suspended in 60 µL of 2× BW buffer. A total of 60 µL of sample was added and rotated at room temperature for 30 min. After incubation, the tubes containing the sample–bead mix were placed on a Magnetic Particle Concentrator for 2 min. The supernatant was discarded, and the beads were re-suspended in Wash1 buffer (1× BW buffer, 0.1% SDS, 0.01% Tween20). This step was repeated three times. For the following two washes, the beads were re-suspended in Wash2 buffer (2× SSC, saline-sodium citrate 0.1% SDS, 0.01% Tween20) instead of Wash1 buffer. The final wash consisted of heating the Wash2 buffer to 65 °C, mixing it with the beads, and incubating the tubes for 2 min at 65 °C while shaking mildly (~300 rpm). Elution of single DNA strands was performed at 95 °C for 3 min by mixing the beads with 25 µL of low TE (Tris-EDTA buffer, 10mM Tris-HCL, 0.1mM EDTA) buffer beforehand. The samples were then amplified for 14 cycles using the Library Preparation Kit polymerase and primer mixes and purified using a 1.5× Agencourt AMPure XP (Beckman Coulter, Brea, CA; USA) purification step according to the manufacturer’s protocols. The samples were run for quality and enrichment control on a High Sensitivity DNA Analysis chip on an Agilent Bioanalyzer (Agilent Technologies). For all samples, a second PEC reaction was preformed using the conditions outlined above except for raising the annealing temperature to 59 °C. We tested the original protocol against a number of modifications and assessed enrichment by loading samples on an HS Agilent Bioanalyzer Chip (Agilent Technologies) where we compared enrichment based on control peak heights and background. The experiments were done in triplicates. This led to the following specific changes from the original protocol:

- The reaction was stopped by adding 10 µL of 0.5 M EDTA to inhibit further polymerase activity as the reaction is cooling down.
- The MinElute purification step was excluded.
- Thirty microliters (30 µL) of Dynabeads MyOne Streptavidin C1 were used in 60 µL 2× BW buffer.
- Washing steps were modified to 3 × 200 µL of 1× BW Buffer at room temperature (RT); 2× 200 µL of 2× SCC (+0.1% SDS, 0.01% Tween 20) at RT; and 1× 2× SCC (+0.1% SDS, 0.01% Tween 20) at 65 °C for 2 min while shaking mildly.
- Elution was done in 25 µL.
- After amplification, the reaction was cleaned up using Ampure XP beads (Agencourt).

In particular, the exclusion of one clean-up step and the more stringent washes led to higher enrichment peaks compared to the original protocol.

### 2.6. Massively Parallel Sequencing

Template preparation was performed using the Ion OneTouch 200 Template Kit v2 following the manufacturer’s protocols (TFS). After recovering the template-positive Ion Sphere Particles (ISPs), the Ion Sphere Quality Control Kit was used to ensure a 10–30% quantity of templated ISPs before continuing to the enrichment stage following the manufacturer’s protocols and using Ion PGM Enrichment Beads (TFS). Sequencing was performed using the Ion PGM Sequencing 200 Kit v2 and Ion 316 or 318 chips (both types either v1 or v2) following the manufacturer’s protocols (TFS).

### 2.7. Data Analysis

For the purpose of this study, all collated DNA sequences were reanalyzed using an in-house analysis pipeline. First, FASTQ sequencing output files were aligned to the revised Cambridge Refeference Sequence (rCRS) [32] using the Burrows-Wheeler Aligner BWA [33] under default parameters. Aligned sequences with a mapping quality above 30 and an aligned-to-soft-clipped-bases ratio of over 75% (except all sequences covering positions 1 or 16569 of the mitogenome) were filtered using python scripts. Base counts at each position of the mitogenome were calculated from the final aligned reads using a self-developed python script. Furthermore, duplicate sequences were marked in each analysis file by using the mark duplicate option of picardtools [34]. The remaining sequences were aligned to human genome assembly hg19, and further processed as described above. Picardtools and SAMtools [35] were used to convert different file formats (BAM, SAM, FASTQ) and read length histograms (RLHs) were created by running the GATK software (v 3.7) [36]. MapDamage 2.0 [37] was used to run a statistical analysis on DNA misincorporation rates.

Analysis of haplotypes and haplogroups was done manually, cross-checking phylogenetic validity using Phylotree [38] and EMMA [39]. Point heteroplasmy (except for positive control) and length heteroplasmy in poly-cytosine tracts were disregarded in this study, including the C-tract between 310 and 316. Differences to the rCRS were called when at least 75% of strands showed the divergent base. Statistical analysis and plots were generated using Microsoft Excel (Microsoft, Redmond, WA, USA) and R [40]. If not indicated otherwise, all statistical calculations concerning the alignments were performed using uniquely aligned read numbers for all samples.

### 2.8. Sanger-Type Sequencing

Sanger-type Sequencing (STS) on hair shaft 1 was performed according to [10], while hair sample 2 was not sequenced with STS due to too low mtDNA content (Table 1).

## 3. Results

**Figure 1:**
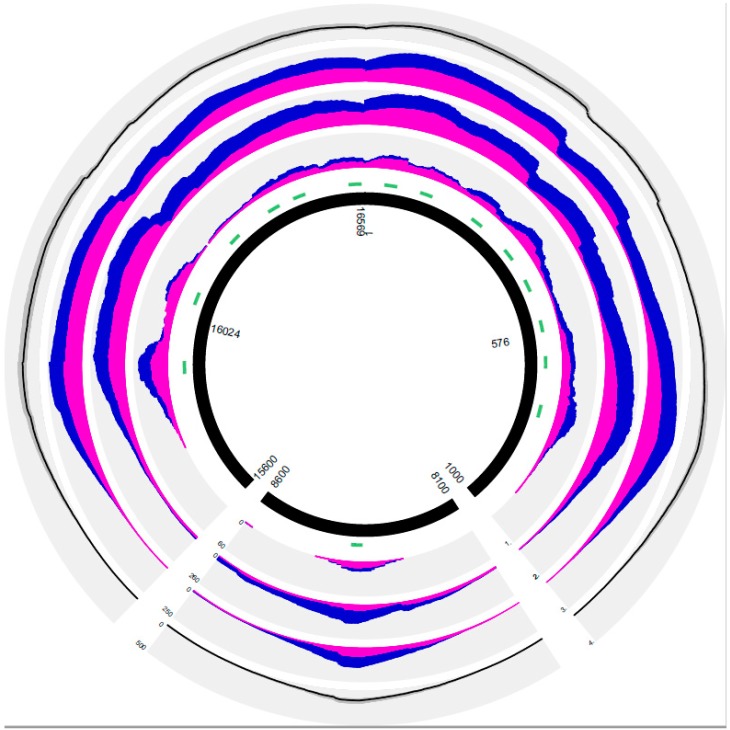
(1) Base count histogram for sample F98-2; (2) base count histogram for sample Hair 1; (3) base count histogram for mean values for all positive controls used in this study; (4) mean coverage values per position (black line) and standard deviation intervals (darker grey interval) across all positive controls. Inner black tiled plot represents the extension of the mitogenome covered with this assay; regions of the mtDNA covered range from 1 to 1000 bp, 8100 to 8600 bp, and 15,600 to 16,569 bp. Green tiles adjacent to the black semicircles depict the PEC biotinylated primer positions. Forward bases are represented in pink and reverse bases in dark blue per mtDNA position along a range of the whole mitochondrial genome.

The 14 PEC primers were designed so that captured mtDNA fragments approximately 70 bp in size could cover the entire CR from positions 16024–576 [32]. However, depending on the actual size of the DNA fragments captured from the DNA extracts, sequencing also went beyond both ends of the CR and covered parts of the coding region (codR) adjacent to the CR (Figure 1). An additional PEC primer was included that targeted the codR around 8342 (Table S1), which we use internally for the quantitation of mtDNA in the casework samples [28]. We further observed an unspecific binding of the primers outside the CR that resulted in sequences aligning to the mtDNA codR. As a consequence, successful PEC experiments resulted not only in CR sequences but also in partial or even full mitogenome sequences, albeit at much lower coverage than in the CR and the region around 8342 (Table 2). This has to be taken into consideration when transferring the method to routine forensic applications, as codR analysis is restricted in some jurisdictions.

### 3.1. Characterization of the PEC Method Based on the Results of Positive and Negative Controls Subsection

The analysis of six different PEC sequencing runs of the positive control extracted from an in-house blood sample allowed some assessment of the reliability and reproducibility of the PEC MPS method (Table 1, Samples 1–6). All positive control PEC sequencing runs resulted in full coverage of the targeted mtDNA CR, with roughly two thirds of total mtDNA reads aligning to the CR (mean 65.14; Table 2). Mean coverage values ranged from 110 to 215× (averaged mean 159.79, mean SD 38.22; Table 2, Figure 1), and yielded CR haplotypes matching those previously derived from this donor by Sanger-type Sequencing (STS, Table S3). All six runs yielded partial mitogenome sequences also matching the STS haplotype in the overlapping regions (Table S3). Gross enrichment in positive control runs, i.e., the relative amount of PEC reads aligned to the mitogenome without filtering for duplicates, amounted to ~3% (averaged mean 3.16, SD 0.4; Table 2), whereas reads aligned to the human genome and unaligned reads amounted on average to 54.67% (SD 7.83) and 42.16% (SD 8.21), respectively. The low SD values (Figure 1) indicate that enrichment remained relatively stable between runs that were performed in the time frame of six months. The amount of unique mtDNA reads relative to total reads ranged from 0.40 to 1.57%, corresponding to 16% and 50% of unique mtDNA reads relative to total mtDNA reads (Table 2).

The negative controls (NPC, NTC, and EX0, Table 2) generally resulted in lower total sequence reads (mean 60,417) compared to those of the positive controls (mean 308,117). In six sequencing runs 0 to 5 (mean 1.5), the reads aligned to the mitogenome with at most one read aligning to the CR (Table 2). These reads, however, all aligned to low complexity repeat regions. Furthermore, the observation of a minimal amount of reads aligning to the target region is in concordance with previous findings [22,41].

### 3.2. General Results of PEC MPS in the Forensic and Solid Tissue Samples

Both forensic hair shaft samples resulted in plausible mtDNA haplotypes using the PEC method (Table S3). Of the 15 solid tissue samples (six teeth and nine bone samples), PEC MPS resulted in five full CR sequences (three teeth, two bones) and five partial CR sequences (three teeth, two bones). Five solid tissue samples, two from the 1 kyrs and three from the 8 kyrs categories, respectively, brought no results (Table 2, Table S3).

Mean strand bias (calculated as the mean of the number of forward reads versus the total number of reads covering in the mitogenome) was relatively uniform (0.3–0.5), with only two samples showing values below 0.2 (Table 2). We noted a slight preference for reverse sequencing reads (Figure 1), an observation that we have made previously with other mtDNA sequencing assays using the Ion PGM (data not shown).

We observed similar mean read lengths (MRL) in the sheared positive control runs (mean 147.08 bp, SD 6.39; Table 2) and comparable values for the forensic samples that yielded useful sequencing results (112–171 bp, Table 2). MRL decreased in the solid tissue samples with increasing age from 136 to 142 bp, at around 142 bp and 96–102 bp in the successful analyses of the 1 kyrs, 2 kyrs, and 8 kyrs categories, respectively (Table 2). Reads mapping to the human genome showed similar read length distributions (Figure S2), whereas non-aligned reads either resulted in smaller MRLs (Table 2) or in read length (RL) distributions skewed towards RLs smaller than 50 bp (Figure S2). In the older samples, we observed increased MRLs (Table 2). These longer reads could stem from exogenous, less degraded DNA, for example from microbial sources [42,43].

### 3.3. PEC MPS Results of the Forensic Samples

PEC MPS on the hair shaft samples resulted in full CR and partial mitogenome haplotypes (Table 1, Table 2, and Table S3). The two hair shaft samples yielded a high relative number of reads aligning to the mitogenome (88.84% and 65.51%, respectively). However, the portion of uniquely mapped mtDNA reads decreased to below 2% in the hair shafts, similar to values found in the positive controls. As the hair shaft samples showed very little non-target background, the limited number of starting molecules in the library amplification seems to have resulted in a higher number of duplicate reads in the final sequencing output.

The 5 cm long hair shaft 1 resulted in a CR haplotype that was fully concordant with the STS results of the corresponding extract (Table S3). Note that STS was performed previous to the PEC analysis in another laboratory by different staff, and only the tube containing the DNA extract was handed to the PEC MPS laboratory. Both hair shaft samples resulted in mtDNA haplotypes typical for the West Eurasian phylogeny (T2b6 and U5b2b, respectively; Table S3) with plausible haplotypes after quality control according to quality criteria established through the EDNAP (The European DNA Profiling Group) mitochondrial DNA database EMPOP [44] that is mandatory in forensic genetics (e.g., [45]).

### 3.4. PEC MPS Results of the Solid Tissue Samples

PEC MPS on the successfully analyzed (*n* = 11) solid tissue samples resulted in total reads between 72,000 and 938,000, with one sample that brought only 7000 reads in total (g52, Table 2). The decreased number of mtDNA target reads in the 1 kyrs category (except g27) could be caused by the generally lower mtDNA yield from these samples. That, and the increase in the proportion of non-aligned reads, may be related to the different environmental conditions those specimens (Volders, Austria) were exposed to - compared to the older samples (Sogamoso, Colombia; Table 1). The successfully typed specimens resulted in 90% to full CR coverage with the exception of two samples, F98E-1 and g121, with 76% and 39% CR coverage, respectively (Table 2). We consistently observed about 10% or more unique mtDNA reads of total mtDNA reads. Lower enrichment values generally correlated with a higher relative number of non-aligned reads (*R* = −0.78; Table 2). Since the PEC assay furthermore targeted a region around position 8342 and extended both ends of the CR, as well as taking into account random non-targeted mtDNA reads from non-removed background DNA, the coverage levels (minimum 2×) of the mitogenome reached up to 70%.

The 1 kyrs old samples from Volders (Austria) resulted in three different haplotypes of West Eurasian provenience, T1a12, H16d, and H11a, respectively (Phylotree, build 17, [38]), with plausible haplotypes after EMPOP quality control [44]. The T1a12 haplotype (g52) was confirmed by STS in the overlapping regions (Table S3) in a different sample from the same individual. Sample g27 yielded an identical mtDNA haplotype using STS from the same DNA extract (Table S3). The PEC-generated haplotypes from the Colombian samples were assigned to haplogroups typically encountered in Native South Americans, i.e., B2d for the 2 kyrs and C1(b) for the 8 kyrs old specimens [46]. Both extracts of the 2 kyrs category (DT08 and DT11-1; Table 1) and one extract of the 8 kyrs category (F98E-1, Table 1) were analyzed with the multiplex PCR-based MT assay that used 162 amplicons of up to 175 bp to target the entire mitogenome. Of these, only sample DT08 resulted in a partial MT mitogenome sequence that matched the PEC results in the overlapping regions (Table S3). A more detailed analysis and interpretation of these observations in the phylogeographic and population genetic context, including additional samples, will be published elsewhere.

The authenticity of the solid tissue sequences was also examined by analyzing the expected cytosine-to-thymine misincorporation rates at the 5′ and 3′ ends [47,48] of the uniquely aligned sequence reads with MapDamage 2.0 [36]. For the solid tissue samples, we observed position-specific probabilities of observing a C->T transition due to post-mortem damage at 5′ ends of the reads above 0.8 on average (SD 0.08, Table S2). In contrast, much lower average probabilities were found in the positive controls (mean 0.26, SD 0.04) and in the forensic samples (mean 0.49, SD 0.06). Similar probabilities were observed for G->A transitions in 3′ read ends (Table S2).

## 4. Discussion

Mitochondrial (mt)DNA is targeted in forensic genetic analyses when evidentiary samples do not contain enough nuclear DNA for conventional STR typing [1,9]. With electrophoretic separation methods and Sanger-type Sequencing (STS), mtDNA typing has typically been restricted to the control region [10] or its two hypervariable segments [5] due to the limited amount of mtDNA contained in these samples (mostly hair shafts and solid tissue samples). With the emergence of Massively Parallel Sequencing (MPS) technologies new possibilities arose for mtDNA typing, and it has been demonstrated that full mitogenome sequences can be generated from 2 cm hair shafts using multiplex PCR-based amplification and sequencing of short (ca. 300 bp) overlapping fragments as well as with shotgun sequencing methods [14]. With this study, we demonstrate that MPS can also be successfully applied in the forensic context to sequence mtDNA from even smaller amounts of and substantially more degraded DNA using a Primer Extension Capture (PEC) methodology that was originally developed for the sequence analysis of Neanderthal mitogenomes [25], thus targeting smaller fragments than PCR-based methods typically do. To the best of our knowledge, this is the first application of PEC to forensically relevant samples. This study is not intended as methodological validation in the sense of forensic accreditation, but presents data from experiments that demonstrate its fitness for the purpose of sequencing extremely degraded DNA with forensic criteria in mind. Since the PEC method is not based on PCR-generated amplicons, as are the majority of established protocols, we particularly considered the use of positive and negative controls, which both have fundamental importance in forensic genetics, as well as suitable guidelines for the interpretation of the resulting sequences.

### 4.1. Positive Controls and PEC Analyses

Anecdotal reports claim that positive controls cannot be used in conjunction with highly sensitive capture hybridization and PEC methods, with the main argument of an elevated risk of cross-contamination. We did not find any written documentation of this statement, and thus treated it as rumor worth addressing in our experimental plan. In each of six PEC runs of forensic and aDNA sample batches we included a positive control, previously extracted from whole blood in a different laboratory and sheared to produce degraded DNA. For library preparation and subsequent library amplification (10 cycles), 1 µL of the sheared control DNA (corresponding to 209,683.60 mtDNA genome equivalents (mtGE)/µL) was used. These steps were performed independently and on different days to the library preparation of any forensic or solid tissue DNA samples. Furthermore, we established a library preparation control for sample batches containing 1 µL of sheared *E. coli* DNA to provide information on successful adapter ligation. Each PEC run also included an enrichment control, containing 10,000,000 copies of a 400 bp fragment targeted by the additional primer at position 8342. After each PEC reaction, this sample showed an elevated peak at around 500 bp on the Agilent HS Chip analysis.

The positive control DNA harbors a rare mtDNA haplotype belonging to haplogroup A10 that is more common in the East Asian and North Asian phylogenies and thus easy to spot as a (minor) contribution in samples of other proveniences (e.g., West Eurasian, Native American). In none of our experiments presented in this study, nor the plethora of unpublished experiments that we have performed with PEC so far, did we encounter a single instance of cross-contamination where this positive would have been involved, either as a sole sequence or as a component of a mixture, although the DNA was used in relatively high amounts (>209,683.60 mtGE/µL). This concerns the different generations of negative controls that were also included in each batch as well as those samples that did not contain enough mtDNA to produce successful PEC results. Based on the generated data, we thus cannot confirm that the application of positive control samples in batches of evidentiary samples, as is routine practice in forensic genetics, would be incompatible with PEC. We do note, however, that utmost care needs to be practiced when preparing libraries for PEC and that the generation of a suitable positive control sample needs to be performed independently and in other laboratory premises.

The PEC results of the positive control sample that was repeatedly analyzed at different times in the course of the presented study (six months) allows some assessment of the reliability and reproducibility of the PEC method. The observed number of total reads and reads aligned to the mtDNA CR and the mitogenome remained stable over the analytical period of six months with low standard deviation. This suggests that the method is stable and reproducible in terms of the quantitative aspect of sequence coverage.

Most MPS output pipelines generate so-called duplicate reads. These reads arise from either PCR amplification or are generated during sequencing in optical sequencing devices (such as the Illumina MiSeq). Duplicates generally do not provide new genuine sequencing information, and are therefore often filtered (except for PCR-based libraries). Duplicate reads can be defined in a variety of ways with the ‘markduplicate option’ of picardtools [49] being a commonly used option. The exact definition can be found in their documentation, but it resorts to comparing read starting positions and base and mapping quality scores. In the positive controls, we observed increasing duplicate reads with increasing sequencing output, suggesting saturation of uniquely targeted molecules. Independent experiments with highly diluted positive control samples spiked with artificial background DNA confirmed that observation (Figure S3). This suggests that total sequencing output could potentially be reduced in future routine applications using PEC MPS, thereby reducing cost without losing forensically relevant information. In the two investigated hair shaft samples that included only minute amounts of mtDNA, we still found a high number of duplicates in the sequencing output, which suggests that modern hair, even when difficult to successfully analyze with PCR mini-amplicon assays (approximately 175 bp amplicons), contains enough short mtDNA fragments for PEC MPS. It has been demonstrated earlier that mtDNA of hair shaft samples can successfully be sequenced via a shotgun approach potentially including even nDNA markers [17]. However, in the absence of comparative data, we expect coverage levels to be higher with the PEC MPS approach due to the targeted nature of the assay.

### 4.2. Regime and Results of Negative Controls

Per batch of forensic/solid tissue samples, a total of four negative controls (NCs) were used. This included the:

(i) extraction blank (EX0) that came with each batch of DNA extracted samples,
(ii) no template control (NTC) that included *E. coli* to increase the complexity of background DNA,
(iii) no primer control (NPC), and
(iv) no template library preparation control (NTC-LP) consisting of a sheared *E. coli* DNA template for monitoring correct adapter annealing.

The NPCs were used to monitor potential contamination during the PEC reaction, and the NTCs served as control for potential cross-contamination during the PEC reaction. Negative controls only showed 1.5 reads aligned to the mitogenome on average, with those reads exclusively aligning to low complexity, repetitive sequence sections. As a consequence, NCs were only sequenced for the first couple of runs to assess background, but were omitted later from the sequencing runs for preserving higher sequencing throughput and cost efficiency.

### 4.3. Selection Criteria for and Results of Samples Included in This Study

The samples included in this study were selected based on the following criteria (Table 1): (a) forensic samples that did not contain enough nuclear DNA for conventional STR analysis, but gave full and reliable mtDNA CR results (Sample 7) as well as too low quantities for STS (Sample 8); (b) DNA extracts from solid tissues with qPCR results well below the threshold of 1000 mtGE that we use internally as a lower bound for promising mtDNA analyses (Samples 9–15); and (c) DNA extracts that did not provide any (Samples 18 and 19) or only partial (Sample 17) results with PCR-based MPS using short amplicons (mitotiling (MT) TFS). The solid tissue samples were selected from three different time periods, 1 kyrs (Samples 9–16), 2 kyrs (Samples 17–18) and 8 kyrs (Samples 19–24), respectively. This allowed for the direct comparison of the PEC data generated in this study with previous STS and MT MPS data.

In the above-mentioned samples that provided either partial or full STS or MT results, we observed identical mtDNA haplotypes in the overlapping regions with PEC MPS. In addition to the confirmatory data obtained with the high quantity positive controls, these results demonstrate concordance between different sequence technologies. For those samples where no direct comparison was possible to independent sequencing results, phylogenetic plausibility checks were performed. The samples were selected so that we would expect West Eurasian lineages for the 1 kyrs category from Volders (Austria) and Native American lineages for the 2 and 8 kyrs categories, which was indeed the case. Quality control metrics that are systematically applied to modern mtDNA data did not suggest the presence of sequence artefacts or data idiosyncrasies. The resulting haplotypes were further not observed in laboratory staff. Furthermore, two 8 kyrs DNA extracts (F98E-1 and F98E-4) stemmed from two different solid tissue sources of the same individual, respectively, and, as expected, produced matching haplotypes.

The PEC MPS method was applied to a forensic case including two hair shafts (5 and 3 cm) that were found at the crime scene in close vicinity to the victim and in causal context with the murder. The victim was excluded as donor of the two hair samples based on morphological considerations. Autosomal STR typing was not performed due to the lack of nuclear DNA (data not shown). The mtDNA CR haplotype (STS) from hair shaft 1 excluded the suspect as donor of that sample. Hair shaft 2 of the same case was not analyzed with STS as the amount of mtDNA determined by qPCR was below the threshold. That left the question open, as to whether the suspect could be excluded as donor of hair sample 2. The judge agreed to test the mtDNA extracted from both hair shafts using the PEC MPS method. Hair shaft 1 yielded concordant results between STS and PEC MPS analysis, thus serving as confirmation between the two different sequencing technologies. The PEC MPS analysis was also successful in hair shaft 2, and resulted in a phylogenetic plausible haplotype. This haplotype was different to the ones observed in hair shaft 1 and the suspect, and thus excluded the suspect also as donor of the second hair sample.

The authenticity of the ancient solid tissue sequences was assessed by analyzing the misincorporation rates at the 5′ and 3′ ends of the sequence reads [47,48]. The most commonly observed post-mortem age-related damage pattern consists of cytosin (C) to uracil (U) deaminations leading to thymin (T) incorporations in subsequent PCR. The C to U conversions are more likely to happen at the ends of the DNA fragments [47,50,51]. Therefore, damage levels can be assessed by elevated C–T misincorporation rates in 5′ and elevated A–G misincorporation rates in 3′ ends. The misincorporation rates observed in our data brought the expected relative values between the modern samples (positive control, forensic samples) and the three different age categories in the solid tissue samples, further corroborating the authenticity of the results obtained with PEC MPS.

One remarkable finding in our study was that input DNA copy numbers did not systematically correlate with different types of success metrics, i.e., number of total or unique mtDNA reads, coverage levels, or mean mtDNA read lengths (MRLs). On the one hand, samples showing 0 quantification results could have contained fragmented DNA with lengths considerably below 143 bp; on the other hand, solid tissue samples with reasonable mtGE/µL content could simply have had too much additional background DNA stemming from other sources hindering successful PEC enrichment. Further experiments using differently sized qPCR target regions, as well as assessing total DNA amounts, could provide further insight into the input DNA and sequencing output dynamics.

Mean mtDNA read lengths for the solid tissue samples were generally higher than expected compared to earlier reports [19,22,26], in which hybridization and/or PEC-based enrichment approaches resulted in smaller MRL values (around 50 bp). We propose the following explanations.

First, all solid tissue samples described in this study were extracted following protocols that come with a greater loss of molecules of a size below 75 bp [19]. After library preparation and each round of PEC enrichment, the samples were checked for their DNA fragment size distribution. We did not observe differences between the cycles, indicating that smaller molecules were already not present after library preparation. In addition to this study, we have also used this PEC assay for the analysis of aDNA samples (1000–2000 years old) that were extracted via another extraction protocol that targeted smaller fragment sizes [19]. In these extracts, we observed an increased portion of read lengths below 100 bp (data not shown). Real-time PCR mtDNA quantitation in those samples indicated an approximately 100-fold surplus of short (67 bp target) over longer fragments (143 bp target). However, since MRLs were still above 50 bp, the assay possibly targeted the few, but longer fragments more efficiently.

Second, smaller fragments could have been excluded due to alignment difficulties using BWA. We therefore filtered unaligned reads for sizes up to 50 bp for several samples that showed a high portion of small, non-aligned reads, and aligned these using small-read aligners such as GMAP [52], bowtie [53], and bowtie2 [54]. These alignments, however, suffered either from too low coverage or too much background noise, depending on the stringency of parameter settings. Furthermore, we calculated sequence complexity values [55] for aligned and non-aligned reads for some of our samples. The results indicate that a large portion of non-aligned reads shows much lower complexity scores, with most of these reads being composed of multiple di- or trimeric repeats (data not shown).

Third, the observed read lengths, at least in the solid tissue samples, could potentially be an indication of possible contamination with modern human DNA. However, all calculations for assessing post-mortem damage patterns clearly showed a substantially different pattern to the sequences obtained from the modern samples (hair shafts and positive controls). Furthermore, there was no evidence of contamination in the sense of mixed reads, and all resulting haplotypes were plausible and concordant in cases where comparisons between samples were possible.

Fourth and finally, amplification bias in the library amplification protocol could have theoretically led to preferential amplification of longer fragments. We used the proprietary commercial primer and polymerase mix for preparing Personal Genome Machine (PGM, TFS) sequencing libraries. If there was systematic bias in amplification, one would expect similar observations in other laboratories. To the best of our knowledge, we did not find any evidence for this thus far. Furthermore, even if some bias was detectable, the enrichment for larger molecules would only be desirable, as longer reads produce more confident alignments with commonly used aligners.

## 5. Conclusions

In this study, we modified a published Primer Extension Capture Massively Parallel Sequencing (PEC MPS) protocol, originally developed for the sequence analysis of the Neanderthal mitogenome, to comply with the requirements of forensic mtDNA typing. The modified and optimized protocol (single tube assay, less mtDNA input required for sequencing, shorter laboratory times) targets the mtDNA control region (CR), however, sequences may expand beyond the CR depending on the quality and quantity of the mtDNA at hand. The PEC MPS protocol was successfully applied to the sequence analysis of two forensically relevant hair shaft samples, one of which gave confirmatory results with conventional Sanger-type Sequence (STS) analyses, whereas the other did not contain enough mtDNA for STS. PEC MPS was further used to analyze 15 solid tissue samples of 1, 2, and 8 kyrs of age, respectively, and resulted in plausible haplotypes in 11 of the 16 samples, including extracts that yielded no results with STS and mitotiling MPS approaches. A direct comparison of the PEC MPS results to STS data was possible in three solid tissue samples and yielded concordant haplotypes, respectively. The PEC-MPS method yielded plausible mtDNA haplotypes in two solid tissue samples that provided no result with PCR-based ‘mini-amplicon’ MPS (mitotiling) that is known to be the most sensitive mtDNA-based sequencing method currently applied in forensic genetics. Special focus was directed on potential contamination issues by using four generations of negative controls, in none of which cross-contamination, carry-over, or spontaneous contamination was observed.

We further propose and have already started experiments on extending the presented approach to target the entire mitogenome in a single tube assay for those laboratories that are not limited by legal restrictions to sequence the coding region [34]. In addition, the effect of different extraction methods on PEC enrichment values, mean read lengths, and on other success metrics needs to be evaluated. Enrichment efficiencies could further be quantified by measuring mtDNA copy number in samples before and after PEC enrichment. Furthermore, in order to eliminate repetitive and low-complexity DNA fragments, different blocking strategies need to be tested in combination with PEC MPS. Lastly, BLAST analysis of long high complexity non-aligned reads could provide information on the composition of exogenous DNA sources.

This study offers experiments and results that serve as proof of principle for the application of PEC MPS in the forensic context. Further work is necessary to translate existing criteria of good laboratory practice in forensic mtDNA genetics [9] to the application of PEC MPS. This involves the definition of minimum criteria for conducting MPS in the forensic arena (e.g., number of positive and negative controls, minimum read coverage, strand bias, redundant sequence coverage, etc.) and appropriate interpretation guidelines to permit validation strategies and finally accreditation of the method in forensic laboratories.

## Figures and Tables

**Table 1:** Description of samples used for this study.

Sample						mtGE/µL	Volume	Analysis Method		
ID	No.	TYPE	AGE	TISSUE	EXTR. METHOD	143 bp	PEC	PEC-CR	MT	STS-CR
**Positive controls**										
PC 1-6	1–6	Control	recent	blood	Qiagen Blood Maxi	~1,700,000	1 µL in 25 µL H_2_O	full	full	full
**Forensic samples**										
Hair 1	7	Forensic	recent	hair	QIAamp DNA Investigator	972	27	full	-	full
Hair 2	8	Forensic	recent	hair	QIAamp DNA Investigator	5	42	full	-	-
**Ancient solid tissue samples**										
g52	9	aDNA	~1 kyrs	molar	PCI	60	30	full	-	partial
g27	10	aDNA	~1 kyrs	molar	SF	NA	25	full	-	full
g43	11	aDNA	~1 kyrs	femur	SF	0	30	full	-	-
g121	12	aDNA	~1 kyrs	femur	PCl	0	30	partial	-	-
g17	13	aDNA	~1 kyrs	costa	PCI	0	30	partial	-	-
g81	14	aDNA	~1 kyrs	tubular bone	PCI	0	28	fail	-	-
g7	15	aDNA	~1 kyrs	humerus	PCI	0	28	fail	-	-
DT08	16	aDNA	~2 kyrs	anterior tooth	SF	149	25	full	partial	-
DT11-1	17	aDNA	~2 kyrs	femur	SF	216	28	full	fail	-
F98E-1	18	aDNA	~8 kyrs	anterior tooth	SF	11,722	28	partial	fail	-
F98E-4	19	aDNA	~8 kyrs	fragment anterior tooth	SF	2005	30	partial	-	-
F98F-2	20	aDNA	~8 kyrs	molar	SF	736	30	partial	-	-
F98A-2	21	aDNA	~8 kyrs	skull fragment	SF	1633	30	fail	-	-
F98B-1	22	aDNA	~8 kyrs	skull fragment	SF	2272	30	fail	-	-
F98C-2	23	aDNA	~8 kyrs	skull fragment	SF	12,543	30	fail	-	-

Type, age, tissue type, extraction method (Extr. Method, SF = SpinFilter; PCI = Phenol Cholorform Isopropapnol Organic Extraction), and qPCR results for a 143 bp mtDNA TaqMan assay are given in columns C–G, followed by the volume of DNA sample extract in µL used for library preparation and subsequent Primer Extension Capture (PEC) analysis (H). Columns I–K provide results of three different analysis methods; CR-PEC = Control Region-Primer Extension Capture; MT = Mitotiling; STS = Sanger-type Sequencing. Full = full CR profile; partial = partial CR profile; fail = insufficient or no data for analysis; - = not analyzed; mtGE = mitochondrial Genome Equivalents.

**Table 2:** Numerical description of sequenced samples. Mean coverage is calculated as the mean number of bases per position covered.

Sample Number		Total Reads	% mtDNA Reads of Total Reads	% Unique mtDNA Reads/Total Reads	No. of unique mtDNA reads	% CR Reads/Total mtDNA Reads	% Human Genome Aligned Reads/Total Reads	% Unaligned Reads/Total Reads	Mean Strand Bias	% Coverage of Whole Mitogenome (min 2×)	% CR Coverage (min 2×)	Minimal CR Coverage	Mean CR Coverage	Mean SD of CR Coverage	Maximal CR Coverage	Minimal RL	Mean RL	SD of Mean RL	Maximal RL	Mean RL of Aligned Reads	Mean RL Other Reads
**Positive controls**																					
**PC 1**	**1**	**102,534**	**3.11**	**1.57**	**1605**	**67.66**	**57.55**	**39.34**	**0.43**	**35.74**	**100.00**	**57**	**110.33**	**27.13**	**171**	**34**	**148.20**	**51.76**	**308**	**134.64**	**56.89**
**PC 2**	**2**	**233,078**	**3.50**	**1.07**	**2495**	**66.33**	**56.08**	**40.42**	**0.40**	**48.96**	**100.00**	**90**	**176.01**	**41.71**	**249**	**30**	**154.69**	**54.66**	**326**	**133.98**	**51.94**
**PC 3**	**3**	**842,114**	**2.40**	**0.40**	**3346**	**61.78**	**38.31**	**59.29**	**0.39**	**70.90**	**100.00**	**112**	**215.50**	**54.49**	**301**	**30**	**150.84**	**53.56**	**331**	**123.71**	**32.74**
**PC 4**	**4**	**125,099**	**3.22**	**1.42**	**1777**	**66.18**	**54.03**	**42.75**	**0.40**	**34.16**	**100.00**	**60**	**114.85**	**25.00**	**168**	**33**	**135.68**	**43.69**	**311**	**122.34**	**50.64**
**PC 5**	**5**	**197,285**	**2.89**	**1.11**	**2191**	**66.77**	**58.97**	**38.14**	**0.40**	**48.30**	**100.00**	**81**	**150.50**	**37.15**	**228**	**30**	**146.81**	**51.57**	**330**	**129.78**	**54.29**
**PC 6**	**6**	**348,593**	**3.87**	**0.85**	**2957**	**62.09**	**63.09**	**33.04**	**0.39**	**60.70**	**100.00**	**108**	**191.57**	**43.81**	**275**	**31**	**146.26**	**47.11**	**316**	**123.06**	**64.72**
**mean**		**308,117.2**	**3.16 (0.4)**	**1.07**	**2395.17**	**65.14**	**54.67 (7.83)**	**42.16 (8.21)**	**0.40 (0.01)**	**49.79**	**100.00**	**84.67**	**159.79 (42.26)**	**38.22**	**232.00**	**31.33**	**147.08 (6.39)**	**50.39**	**320.33**	**127.92**	**51.87**
**Forensic samples**																					
**Hair 1**	**7**	**256,877**	**88.84**	**1.39**	**3565**	**49.14**	**2.05**	**9.11**	**0.36**	**78.77**	**100.00**	**108**	**183.62**	**39.05**	**258**	**30**	**139.82**	**58.87**	**333**	**114.94**	**39.28**
**Hair 2**	**8**	**346,147**	**65.51**	**0.24**	**819**	**44.93**	**5.58**	**28.91**	**0.42**	**54.13**	**100.00**	**2**	**26.85**	**10.28**	**49**	**32**	**112.60**	**38.72**	**241**	**119.60**	**43.63**
**Ancient solid tissue samples**																					
**1 kyrs**																					
**g52**	**9**	**7230**	**14.18**	**8.40**	**607**	**78.25**	**44.04**	**41.78**	**0.15**	**10.40**	**100.00**	**17**	**46.16**	**17.93**	**98**	**31**	**138.57**	**44.52**	**288**	**130.90**	**58.46**
**g27**	**10**	**829,116**	**0.38**	**0.09**	**749**	**60.88**	**54.77**	**44.85**	**0.42**	**35.95**	**100.00**	**21**	**45.29**	**14.97**	**91**	**35**	**138.76**	**49.88**	**309**	**136.47**	**80.81**
**g43**	**11**	**72,886**	**1.38**	**0.84**	**609**	**57.96**	**36.64**	**61.98**	**0.46**	**31.36**	**100.00**	**12**	**33.51**	**11.17**	**61**	**33**	**142.22**	**53.96**	**325**	**141.97**	**115.87**
**g121**	**12**	**88,249**	**0.06**	**0.04**	**37**	**48.65**	**1.00**	**98.95**	**0.53**	**6.73**	**39.13**	**1**	**1.95**	**0.96**	**4**	**70**	**136.30**	**37.11**	**283**	**131.08**	**121.58**
**g17**	**13**	**185,894**	**0.03**	**0.02**	**44**	**68.18**	**0.33**	**99.64**	**0.24**	**10.17**	**94.56**	**1**	**3.08**	**1.48**	**7**	**32**	**141.91**	**58.50**	**279**	**112.27**	**119.91**
g81	14	317,785	0.00	0.00	8	0.00	3.50	96.50	0.00	0.00	0.36	0	0.00	0.00	0	48	188.38	91.77	290	95.64	111.02
g7	15	317,487	1.33	0.03	90	51.11	2.30	96.37	0.47	8.47	63.19	1	4.35	2.76	10	52	132.80	43.99	279	133.24	100.04
**2 kyrs**																					
**DT08**	**16**	**540,060**	**3.41**	**0.56**	**3048**	**56.53**	**51.42**	**45.18**	**0.39**	**71.80**	**100.00**	**59**	**171.61**	**47.51**	**253**	**31**	**142.85**	**53.37**	**311**	**130.19**	**114.19**
**DT11-1**	**17**	**201,917**	**16.09**	**0.91**	**1829**	**53.31**	**45.88**	**38.03**	**0.40**	**68.45**	**100.00**	**37**	**96.37**	**27.00**	**145**	**31**	**142.08**	**56.34**	**315**	**138.45**	**94.66**
**8 kyrs**																					
**F98E-1**	**18**	**879,757**	**0.06**	**0.01**	**69**	**55.07**	**0.03**	**99.91**	**0.52**	**8.05**	**76.29**	**1**	**2.99**	**1.60**	**8**	**37**	**96.45**	**40.30**	**342**	**104.01**	**142.54**
**F98E-4**	**19**	**532,948**	**0.14**	**0.04**	**206**	**52.91**	**0.11**	**99.76**	**0.49**	**10.52**	**96.43**	**1**	**8.10**	**4.22**	**18**	**38**	**100.65**	**34.30**	**244**	**94.57**	**124.41**
**F98F-2**	**20**	**534,508**	**0.84**	**0.06**	**318**	**61.32**	**0.90**	**98.26**	**0.47**	**12.36**	**99.20**	**1**	**14.42**	**6.67**	**32**	**37**	**102.75**	**37.63**	**246**	**108.52**	**128.40**
F98A-2	21	73	0.00	0.00	0	0.00	0.00	100.00	0.00	0.00	0.36	0	0.00	0.00	0	0	0.00	0.00	0	NaN	173.97
F98B-1	22	451	0.22	0.22	1	100.00	0.00	99.78	1.00	0.00	0.00	1	1.00	0.00	1	98	98.00	NA	98	NaN	125.79
F98C-2	23	910	1.21	0.33	3	100.00	0.00	98.79	0.38	0.88	12.92	2	2.70	0.46	3	101	130.33	25.40	145	NaN	95.68
**Negtive Controls**																					
NPC		4146	0.12	0.12	5	20.00	75.01	24.87	0.00	0.40	0.00	1	1.00	0.00	1	76	152.60	54.00	226	151.06	77.85
NTC-LP		48,396	0.00	0.00	0	0.00	8.41	91.59	0.00	0.00	0.36	0	0.00	0.00	0	0	0.00	0.00	0	162.85	40.23
NTC-LP		37,769	0.00	0.00	1	100.00	18.35	81.65	1.00	0.00	0.00	1	1.00	0.00	1	135	135.00	NA	135	142.74	27.95
NTC_LP		81,476	0.01	0.00	2	50.00	9.99	90.01	1.00	0.00	0.00	1	1.00	0.00	1	66	98.50	45.96	131	134.78	26.94
EX0		31,886	0.00	0.00	0	0.00	24.81	75.19	0.00	0.00	0.36	0	0.00	0.00	0	0	0.00	0.00	0	126.05	60.15
EX0		158,827	0.00	0.00	1	0.00	1.35	98.65	0.00	0.00	0.36	0	0.00	0.00	0	70	70.00	NA	70	127.15	42.96
mean		60,417	0.02	0.02	2	28.33	22.99	76.99	**0.33**	**0.07**	**0.18**	**0.50**	**0.50**	**0.00**	**0.50**	**57.83**					

SD = Standard Deviation; RL = Read Length; NPC = no primer control; PC = positive control; NTC = no template control; LP = library preparation blank; EX0 = extraction blank.

## Supplementary Materials

The following are available online at www.mdpi.com/2073-4425/8/10/237/s1. Table S1: Details of primers designed for PEC MPS; Table S2: Position-specific probability of observing a C->T or a G->A change due to post-mortem damage. Positions include the first 12 (1–12) and last 12 (from –12 to –1) bases of reads; Table S3: Overview of PEC MPS haplotypes generated in this study and Sanger/mitotiling results. Haplogroup based on Phylotree, build 17. C-runs and 315.1C were disregarded in PEC analyses. Results from same sample analyzed with different methods are coloured in grey-scale. Figure S1: Coverage plots per sample for mtDNA control region sequencing by PEC-MPS. Inner black tiled plots represent the extension of the mitogenome covered with this assay, regions of the mtDNA covered range from 1 to 1000 bp, 8100 to 8600 bp, and 15,600 to 16,569 bp. Green tiles adjacent to the black semi circles depict the PEC biotinylated primer positions. For each sample forward (pink) and reverse (blue) reads are plotted. Samples are divided into 4 subgroups: A: Forensic samples (Hair shaft 1 and 2), B: Volders samples (sample 1: g52, sample 2: g43, sample 3: g121, sample 4: g17, sample 5: g27), C: Colombia 2 kyrs samples (sample 1: DT08-1, sample 2: DT11-1 ), and D: Colombia 8 kyrs samples (sample 1: F98E-1, sample 2: F98E-4 and sample 3: F98F-2); Figure S2: Read length histograms of each tested sample for reads aligned to the mitogenome, human genome, and the unaligned reads (rest); Figure S3: (1) reads after PEC performed on a positive control sample diluted to a total of ~100 mtGE. (2–4) reads after PEC performed on three replicates of a positive control sample diluted to a total of ~100 mtGE spiked with 5 ng of sheared *E. coli* DNA. Forward reads are plotted in pink, reverse reads in dark blue.
